# Political Astuteness in Nurse Leadership: A Concept Analysis

**DOI:** 10.1155/jonm/7258276

**Published:** 2026-05-28

**Authors:** Namadzavho Joyce Muswede, Vhothusa Edward Matahela

**Affiliations:** ^1^ Professional Practice Department, South African Nursing Council, Pretoria, Gauteng, South Africa; ^2^ Department of Health Studies, University of South Africa, Pretoria, Gauteng, South Africa, unisa.ac.za

**Keywords:** governance, healthcare reform, nurse leadership, nursing education, policy advocacy, political astuteness, political competency, strategic decision-making

## Abstract

**Aim:**

To conduct a concept analysis of political astuteness in nurse leadership, clarifying its attributes, antecedents and consequences.

**Design:**

Concept analysis.

**Data Sources:**

Literature published between 1990 and February 2025 was searched in EBSCOhost, Emerald, ProQuest, Sabinet, SAGE and ScienceDirect.

**Methods:**

Walker and Avant’s (2019) eight‐step concept analysis framework was used to identify defining attributes, antecedents, consequences and empirical referents. Model and contrary cases were constructed to illustrate the presence and absence of the concept.

**Results:**

Analysis of 24 included sources identified defining attributes of political astuteness in nurse leadership, including strategic awareness, policy acumen, advocacy, stakeholder engagement and ethical reasoning. Antecedents included policy knowledge, emotional intelligence, governance exposure and experience of political environments. Reported consequences in the literature included stronger strategic and ethical decision‐making, greater capacity for stakeholder alignment and increased potential to engage in policy and organisational influence.

**Conclusion:**

This concept analysis suggests that political astuteness is a relevant leadership capability in nurse leadership, particularly in contexts requiring policy engagement, stakeholder alignment and navigation of complex organisational environments. Clarifying the concept may support more coherent scholarship, leadership development and future empirical work in this area.

**Implications for the Profession:**

This study contributes conceptual clarity to political astuteness in nurse leadership and offers a basis for considering how political capability may be addressed in leadership development and nursing education. The findings may also inform future work on how nurse leaders are prepared for policy engagement, organisational influence and system‐level leadership.

**Reporting Method:**

A PRISMA 2020 flow diagram was used to report the literature identification and selection process within this concept analysis.

**Patient or Public Involvement:**

This study was a concept analysis; therefore, no patient or public involvement in its design, conduct or reporting was required.

## 1. Introduction

Political astuteness is increasingly recognised as an important leadership capability for nurse leaders who work across policy processes, organisational politics and interprofessional relationships. In this paper, the concept denotes a broader capability than interpersonal influence alone: it combines contextual reading, strategic judgement, stakeholder alignment and ethically informed action in settings where power, resources and priorities are contested [1, 2]. Although political astuteness has been developed mainly in public leadership scholarship, its specific meaning for nurse leadership remains insufficiently clarified.

The nursing literature often places political astuteness alongside related constructs such as political skill, political awareness, political acumen, political competence, political efficacy and policy advocacy. These constructs are useful but not equivalent. Political skill foregrounds interpersonal influence and networking; political awareness and acumen emphasise sensitivity to power and judgement; political competence, efficacy and advocacy focus on preparedness, confidence and action in policy participation. Political astuteness is broader because it includes reading the wider context, working across stakeholders and using strategy ethically across organisational and policy arenas [1–4].

Without this distinction, political astuteness remains difficult to teach, develop or study in nurse leadership. A concept analysis is therefore needed to clarify its defining attributes, antecedents, consequences and empirical referents and to provide a more coherent foundation for nursing leadership scholarship, education and professional development.

## 2. Background

Political astuteness is most useful in this analysis as a leadership capability that helps nurse leaders interpret complex policy and organisational environments, identify interests and power relationships and act strategically while remaining accountable to professional and public values. Health leadership studies link political astuteness with stakeholder mapping, coalition building, strategic framing, timing and the management of informal power in major change processes [1, 2, 5, 6]. Nursing‐specific work similarly shows the relevance of political preparation and policy engagement for nurse leaders, while also indicating that many nurses and nurse leaders remain underprepared for sustained participation in policy and governance [7–9].

To avoid conflation, this analysis treats political astuteness as distinct from, but related to, several adjacent constructs. Political skill refers mainly to interpersonal influence, social astuteness, networking ability and apparent sincerity [3, 4]. Political competence and political efficacy are more closely linked to knowledge, confidence and participation in policy or political processes [10, 11]. Policy advocacy refers to action directed at influencing policy decisions or public priorities [12, 13]. Political astuteness draws on these capacities but integrates them within a wider leadership capability for reading context, aligning stakeholders, timing action and exercising ethical strategic influence across organisational and system arenas [1, 2, 5].

Such differentiation matters because nurse leaders often see how policy and governance decisions shape staffing, service delivery, quality and equity, yet formal clinical expertise alone does not secure influence in decision‐making spaces [8, 12, 13]. Political astuteness points to the additional capability required to interpret formal and informal systems of power, work with competing interests, build credible alliances and frame nursing concerns in ways that can be heard within organisational and policy processes.

Accordingly, this concept analysis focuses on political astuteness as a multidimensional leadership capability rather than as a synonym for political skill, awareness or advocacy. Clarifying the concept can support more precise use in nurse leadership scholarship and provide a cautious basis for future educational and empirical work.

## 3. Method

This study used Walker and Avant’s [14] eight‐step concept analysis method to examine political astuteness in nurse leadership. Concept analysis is used in nursing scholarship to clarify the meaning of concepts, distinguish them from related terms and support theoretical development and future empirical inquiry [15]. Walker and Avant’s method was selected because it provides a structured process for identifying defining attributes, antecedents, consequences, model and contrary cases, and empirical referents of a concept within a specific disciplinary context. In this study, the analysis followed Walker and Avant’s eight steps: selecting the concept, determining the purpose of the analysis, identifying uses of the concept, determining defining attributes, constructing a model case, constructing a contrary case, identifying antecedents and consequences and identifying empirical referents.

### 3.1. Search Strategy and Study Selection

A literature search was conducted between October 2024 and February 2025 across EBSCOhost, Emerald, ProQuest, Sabinet, SAGE and ScienceDirect. The search covered literature published between 1990 and February 2025. This time frame was selected to capture early work on political skill and related leadership constructs, the later development of political astuteness in public sector and health leadership scholarship, and contemporary work relevant to nurse leadership.

Search terms were developed from the focus of the analysis and combined using Boolean operators. Core terms included ‘political astuteness’, ‘nurse leaders’, ‘political skill’, ‘policy advocacy’ and ‘policy influence’. A keyword‐based strategy was used across databases to ensure consistency in retrieval across platforms with differing indexing systems.

Publications were eligible for inclusion if they were published in English, available in full text and explicitly addressed political astuteness in relation to nurse leadership or provided conceptually relevant discussion that informed its definition, attributes, antecedents, consequences or empirical referents. Publications were excluded if they lacked sufficient conceptual or methodological details to inform the analysis, or if they did not meaningfully address political astuteness within leadership, policy or governance contexts relevant to nursing. English language publications were included because the analysis required close interpretation of conceptual definitions, theoretical nuance and contextual meaning. Full‐text access was required to support detailed extraction and interpretation of the concept.

Two authors independently screened titles and abstracts and then reviewed potentially eligible full‐text articles. Disagreements were resolved through discussion and consensus. No automation tools were used in the screening process. A total of 2223 records were identified. After removal of 1979 duplicates, 244 records remained for screening. Following title and abstract screening, 43 reports were sought for retrieval and assessed for eligibility. Nineteen reports were excluded because they were not conceptually or methodologically relevant to political astuteness. A total of 24 sources met the inclusion criteria and were included in the concept analysis.

**FIGURE 1:**
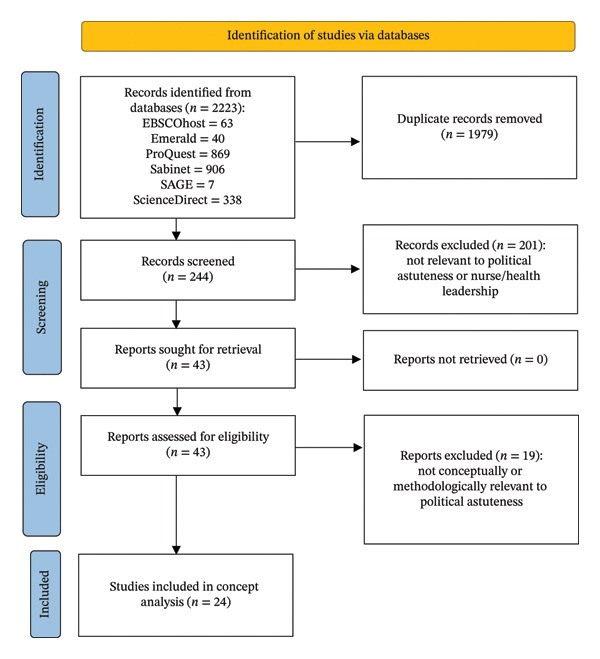
PRISMA flowchart of included articles.

To enhance transparency, the identification and selection process is presented in a PRISMA 2020 flow diagram (Figure 1). In this study, the flow diagram was used to report the search and screening process only; the conceptual analysis itself remained guided by Walker and Avant’s framework.

The included studies are presented in Table 1.

**TABLE 1:** Studies identified for concept analysis of political astuteness (*n* = 24).

Authors, year	Study title	Country	Discipline	Article type/methodology
[16]	Political astuteness as an aid to discerning and creating public value	United Kingdom, Australia	Public Administration, Leadership	Conceptual/theoretical paper (narrative literature review)
[17]	Barriers to and Facilitators of Nurses’ Political Participation in Ghana	Ghana	Nursing	Cross‐sectional survey with a quantitative approach
[18]	Contested Practice: Political Activism in Nursing and Implications for Nursing Education	Canada	Nursing Education	Qualitative exploratory study, using comparative life history methodology
[19]	Political Astuteness of Baccalaureate Nursing Students Following an Active Learning Experience in Health Policy	United States	Nursing Education, Public Health	Quantitative study (pre–post‐test design using the Political Astuteness Inventory—PAI)
[20]	Developing Political Astuteness: A Leadership Coaching Journey	United Kingdom	Educational Leadership, Coaching	Conceptual/theoretical paper (narrative case study approach)
[21]	Civic knowledge and self‐reported political astuteness of academic nurse educators in the United States	United States	Nursing Education	Descriptive cross‐sectional survey
[22]	Leadership with Political Astuteness for Public Servants: And Why It Matters	United Kingdom	Public Administration, Leadership	Conceptual/theoretical paper (narrative review and empirical synthesis)
[1]	Leading with Political Astuteness: A Study of Public Managers in Australia, New Zealand, and the United Kingdom	Australia, New Zealand, United Kingdom	Public Administration, Leadership	Mixed‐methods study (survey, focus groups and interviews)
[23]	Public Value and Political Astuteness in the Work of Public Managers: The Art of the Possible	Australia, New Zealand, United Kingdom	Public Administration, Leadership	Mixed‐methods study (survey, interviews, conceptual analysis)
[24]	Leadership for Public Value: Political Astuteness as a Conceptual Link	United Kingdom, Netherlands	Public Administration, Leadership	Conceptual/theoretical paper (narrative literature review)
[25]	‘It’s Every Breath We Take Here’: Political Astuteness and Ethics in Civil Service Leadership Development	United Kingdom	Public Administration, Leadership	Qualitative study (survey and interviews with senior civil servants)
[26]	Exploring the Business of Urology: Influence Management and Political Skills	Canada	Urology, Healthcare Leadership	Conceptual/theoretical paper (narrative literature review)
[27]	Preparing Nurse Leaders for 2020	United States	Nursing	Review type, literature review with expert opinion
[28]	Factors Influencing Political Self‐Efficacy and Political Astuteness in Undergraduate Nurse Educators	United States	Nursing Education	Quantitative study (descriptive cross‐sectional survey design)
[29]	Dancing on Ice: Leadership with Political Astuteness by Senior Public Servants in the UK	United Kingdom	Public Administration, Leadership	Qualitative study (semi‐structured interviews with senior public servants)
[30]	Political Astuteness: Informing Education for Advocacy and Efficacy	United States	Nursing and Health Policy	Quantitative study (pretest/post‐test survey design using the Political Astuteness Inventory—PAI)
[7]	The Level of Political Astuteness in Nursing Leaders: A Baseline Assessment	United States	Nursing	Cross‐sectional survey
[31]	Changes in Political Astuteness After a Health Systems and Policy Course	United States	Nursing	Quantitative study, using a pre‐post design
[32]	New Development: Picking a Way Through Minefields—Leadership with Political Astuteness for Senior Police	United Kingdom	Public Administration, Policing, Leadership	Conceptual/theoretical paper (narrative literature review, policy analysis)
[8]	Factors that Act as Facilitators and Barriers to Nurse Leaders’ Participation in Health Policy Development	Kenya, Uganda, Tanzania	Nursing	A Delphi survey that included expert panellists, iterative rounds, statistical analysis and consensus building
[33]	Development of Resources to Promote Nurse Anesthetist Engagement in Policy Advocacy	United States	Nursing (Nurse Anesthesia)	Quantitative study (pre–postsurvey design)
[5]	Healthcare Leadership with Political Astuteness (HeLPA): A Qualitative Study of How Service Leaders Understand and Mediate the Informal ‘Power and Politics’ of Major Health System Change	United Kingdom	Healthcare Leadership, Public Administration	Qualitative study (interviews, ethnography, systematic review of reviews, coproduction workshops)
[6]	Healthcare Leadership with Political Astuteness and its Role in the Implementation of Major System Change: The HeLPA Qualitative Study	United Kingdom	Healthcare Leadership, Public Administration	Qualitative study (systematic review, interviews, case studies, codesign workshops)
[34]	Acquiring and Developing Healthcare Leaders’ Political Skills: An Interview Study with Healthcare Leaders	United Kingdom	Healthcare Leadership	Qualitative research (interview study)

## 4. Results

### 4.1. Step 1: Select the Concept

Political astuteness was selected because it captured a broader leadership capability than several related constructs found in the reviewed literature. Political skill emphasises interpersonal influence and networking; political awareness and acumen emphasise judgement about power; political competence and efficacy emphasise knowledge, confidence and participation in policy processes. Political astuteness, by contrast, was most consistently associated with reading people and context, aligning stakeholders and coalitions, timing strategic action and exercising direction across organisational and system settings [1–4, 10].

The concept was therefore selected because it offers a more suitable frame for examining how nurse leaders interpret, navigate and influence complex governance and policy environments without reducing leadership to interpersonal influence or policy participation alone.

### 4.2. Step 2: Determine the Purpose of the Analysis

The purpose of the analysis was to clarify the meaning of political astuteness in nurse leadership by identifying its defining attributes, antecedents, consequences and empirical referents. In doing so, the analysis sought to distinguish the concept from adjacent terms and to provide a clearer conceptual basis for future scholarship in nursing leadership. No prior concept analysis focused specifically on political astuteness in the nursing profession was identified in the reviewed literature.

### 4.3. Step 3: Identify Uses of the Concept

Across the reviewed literature, political astuteness was used primarily in relation to leadership within nursing, health systems and public sector settings. In nursing and health leadership contexts, it referred to the capacity of nurse leaders to interpret political environments, recognise stakeholder interests, build alliances and influence decisions across organisational and policy arenas. These uses were identified across a range of settings, including acute and primary care services, educational institutions, professional associations, regulatory structures and broader health policy environments.

Within nursing, the concept has been used to describe how nursing leaders assess policy opportunities, frame issues strategically and engage in reform‐oriented leadership [7, 9]. It is also reflected in interprofessional coalition building, where healthcare leaders work across professions and services to secure support for change initiatives [5]. At the organisational level, political astuteness is associated with micropolitical work such as reading informal power relations, negotiating trade‐offs and sustaining legitimacy within constrained authority structures [6, 34]. Nursing education literature further links political astuteness with policy learning and political preparation among students and educators [19, 21, 31].

Public leadership literature was used selectively to clarify transferable elements, particularly multilevel influence, stakeholder alignment and ethical action in shared‐power contexts [1, 23, 25]. However, nursing and health leadership literature remained the primary anchor for interpreting the concept in this analysis.

### 4.4. Step 4: Define Attributes

Attributes are the characteristics that repeatedly appear in the literature as central to a concept and that help distinguish it from related constructs [14]. In this analysis, political astuteness in nurse leadership was understood as a leadership capability involving the capacity to read context, interpret stakeholder interests, act strategically and exercise judgement in politically and organisationally complex settings.

Across the reviewed literature, defining attributes were synthesised as contextual and strategic awareness, policy acumen, stakeholder engagement, advocacy‐oriented influence, ethical reasoning and judgement under uncertainty [1, 2, 4–6]. These attributes describe nurse leaders who can recognise the organisational and policy landscape, understand how formal and informal decisions are shaped, align relationships across stakeholders and act in ways that balance strategic feasibility with professional and public values.

Several enabling features appeared repeatedly but were treated as supporting rather than defining attributes. These included interpersonal intelligence, negotiation and diplomacy, communication, networking ability, adaptability and problem‐solving orientation [2–4, 34]. On their own, these features may indicate political skill or general leadership capability; they become expressions of political astuteness when integrated with contextual reading, strategic timing and ethical system‐level influence.

Collectively, the findings support political astuteness as a multidimensional leadership capability with strategic, contextual, relational and ethical dimensions. The concept overlaps with political skill, awareness, acumen, competence, efficacy and advocacy but is not reducible to any one of them. Its distinctive contribution lies in integrating these elements into a broader capacity to read, navigate and influence organisational and policy environments in nurse leadership [1–3, 5].

### 4.5. Step 5: Identify Model Case

Following Walker and Avant [14], researchers developed a constructed model case as a composite scenario created by the authors to demonstrate the defining attributes identified in Step 4 of the analysis. The vignette, represented by the hypothetical example of Dr Thembeka Mkhize, is illustrative rather than evidentiary and does not depict a real individual.

A team of highly skilled nephrology nurses faced restrictive fee‐for‐service policies that limited their scope of practice and compromised patient access to specialised renal care. As team leader, Dr Thembeka Mkhize recognised that change required more than technical expertise. She first mapped the policy landscape, distinguishing between administrators implementing legislation and policymakers with authority to amend it. She led her team in developing an evidence‐based policy brief that combined clinical data, economic arguments and patient outcomes, framing the issue as a public access and health system benefit rather than a narrow professional demand.

Dr Mkhize strategically established coalitions with professional associations, patient groups and legal advisors to amplify nurses’ voices. She led timed engagements with relevant key stakeholders and policy review cycles. Anticipating resistance, she responded through diplomatic and persistent negotiation, acknowledging funders’ concerns while presenting well‐supported counterarguments. These efforts contributed to amendments that expanded reimbursable services in nephrology care and also created momentum for wider reform affecting nurse specialists.

This scenario constitutes a model case because it demonstrates the defining attributes of political astuteness in nurse leadership, including contextual and systems awareness, strategic framing, stakeholder alignment, advocacy, ethical judgement and skilful engagement with decision‐makers.

### 4.6. Step 6: Identify Contrary Case

Contrary cases serve as distinct examples that illustrate instances where the concept is absent or does not apply [14]. The following illustrates a contrary case, which has been amalgamated from qualitative examples in the literature:

Ms Badosi, a senior nurse leader in a provincial health department, was tasked with implementing a policy affecting the care of a vulnerable population. Rather than reviewing the legislation, budget allocations and readiness reports that shaped the policy context, she proceeded with immediate implementation without considering alternative options or recognising major resource and capacity constraints. She cancelled planned meetings with key stakeholders; did not consult community leaders, district health councils or professional associations; and declined invitations from patient advocacy groups and interprofessional forums.

When frontline nurses and clinic managers raised concerns about transport, chronic understaffing and cold‐chain limitations, she dismissed these as resistance to change and enforced departmental circulars rigidly without adapting implementation to local realities. The rollout subsequently faltered: supply chains broke down, clinics missed reporting deadlines, staff lacked adequate preparation and resources, and service disruptions attracted negative media attention, ultimately leading to suspension of the policy. This scenario constitutes a contrary case because the defining attributes of political astuteness are absent. The nurse leader demonstrates little contextual or systems awareness, does not engage stakeholders or build alliances, fails to act strategically in relation to timing and implementation conditions and neglects the ethical and equity dimensions of leadership in a politically and organisationally complex setting.

### 4.7. Step 7: Identify Antecedents and Consequences

#### 4.7.1. Antecedents

Antecedents are events, conditions or capacities that precede the emergence of a concept [14]. In this analysis, political astuteness appeared to depend on both individual capabilities and contextual opportunities. Individual antecedents included policy knowledge, strategic awareness, ethical reasoning, emotional intelligence and previous exposure to governance or politically complex environments [1, 2, 35].

These antecedents span cognitive, emotional and behavioural domains. Cognitively, nurse leaders require policy literacy, analytical judgement and awareness of organisational dynamics. Emotionally, they require the ability to read relational cues and regulate their responses in politically sensitive situations. Behaviourally, prior opportunities to network, communicate strategically and participate in committees, coalitions or policy forums may strengthen the conditions through which political astuteness develops [34, 35].

External antecedents include organisational culture, leadership support, governance structures and formal opportunities for nurses to participate in decision‐making [6, 8]. Regulatory frameworks, policy climates and public expectations also shape whether political activity by nurse leaders is perceived as legitimate, possible and professionally valued [2, 22].

#### 4.7.2. Consequences

Consequences are outcomes that follow the emergence of a concept [14]. In this analysis, the literature suggested possible consequences at individual, organisational and policy levels. Because much of the evidence is conceptual, descriptive or self‐reported, these consequences should be interpreted as plausible and literature‐supported rather than as established causal effects.

At the individual level, political astuteness may support more context‐sensitive strategic and ethical judgement. Politically astute leaders are described as better able to balance professional values, organisational priorities and political realities when making decisions in contested settings [23, 25]. Such practice may also strengthen credibility and legitimacy in policy or governance forums.

At the organisational and health system level, political astuteness may support policy implementation and service change by helping leaders anticipate resistance, frame proposals strategically and broker relationships across professional and organisational boundaries [5, 6]. In nursing contexts, this could contribute to stronger nursing representation in decision‐making, improved stakeholder buy‐in and organisational climates more receptive to nursing‐led change; however, these outcomes require further empirical testing.

At the policy, societal and educational levels, political astuteness may strengthen the visibility and influence of nursing in public debate and formal governance processes. By understanding and negotiating competing interests, nurse leaders may be better positioned to contribute to feasible policy options and to mentor future nurses as policy actors [12, 13, 24].

### 4.8. Step 8: Define Empirical Referents

Empirical referents are observable indicators that help identify a concept in practice [14]. For political astuteness, existing measurement options remain limited. The Political Astuteness Inventory (PAI), originally developed by Clark [36] and later adapted in nursing research by Primomo [31], is the most frequently cited tool linked to the term. However, the PAI primarily reflects political awareness, involvement and selected advocacy behaviours, whereas this analysis conceptualises political astuteness more broadly as a strategic, contextual, relational and ethical leadership capability.

The PAI includes items on policy knowledge, political engagement behaviours, committee involvement and advocacy‐related attitudes. It classifies respondents across levels of political awareness and involvement [31, 36, 37]. Although the instrument has been useful in nursing education and policy preparation studies, it should not be treated as a comprehensive measure of the broader concept described in this analysis. Rather, it offers partial evidence of some cognitive and behavioural dimensions.

Other instruments measure related, but not identical, constructs. The Political Skill Inventory captures social astuteness, interpersonal influence, networking ability and apparent sincerity and is therefore most relevant to the relational and interpersonal dimensions of political astuteness [3]. The Political Competence Scale for Nurses assesses political knowledge, efficacy, interaction and activity [38], while political efficacy and participation measures capture confidence and engagement in political processes [11]. These tools can function only as proxy indicators for selected attributes; none fully captures the contextual, strategic and ethical integration identified in this concept analysis.

Behavioural referents may include documented participation in policy forums, authorship of policy briefs or submissions, leadership in professional coalitions, evidence of stakeholder mapping and records of negotiated organisational or policy change. These indicators may help researchers and educators observe selected manifestations of political astuteness, but they require careful contextual interpretation. The current literature therefore supports cautious use of existing tools and indicators, while highlighting the need for future work to develop and test conceptually aligned measures.

## 5. Discussion

This concept analysis clarifies political astuteness in nurse leadership as a multidimensional leadership capability that extends beyond political awareness, political skill or policy advocacy alone. Across the reviewed literature, it referred to the ability to read context, understand stakeholder interests, exercise strategic judgement and act ethically within organisational and policy environments where power and priorities are contested. It is therefore best understood as an integrative leadership capability for complex health systems, rather than as a single interpersonal or advocacy behaviour [1, 2, 5].

The main contribution of the analysis is conceptual differentiation. Political skill foregrounds interpersonal influence and networking; political competence and efficacy foreground knowledge, confidence and participation; policy advocacy foregrounds action directed at policy change. Political astuteness overlaps with these constructs but brings them together through a wider concern with context, timing, stakeholder alignment and ethically informed strategy. This distinction is important because imprecise use of the term may weaken leadership scholarship and make it difficult to design coherent educational or developmental interventions [1–4, 10].

The analysis also shows practical relevance, but within cautious limits. The literature suggests that politically astute nurse leaders may be better positioned to work in contexts characterised by competing interests, formal and informal power structures and implementation uncertainty. However, the evidence base does not yet support strong causal claims about outcomes. Broader nursing literature on political and policy engagement shows that participation remains uneven and is constrained by organisational, professional and contextual barriers [7, 8, 39–41].

The empirical referents remain the least developed aspect of the concept. The PAI and related instruments provide useful starting points but should be treated as partial or proxy indicators, not as comprehensive measures of political astuteness in nurse leadership. Future methodological work should examine whether existing tools can be adapted or whether a new instrument is needed to capture the contextual, ethical, strategic and relational integration identified in this analysis [3, 31, 36, 38].

Collectively, the findings suggest that political astuteness may help explain how nurse leaders engage with policy, negotiate organisational complexity and exercise influence when formal authority alone is insufficient. It also offers a potentially useful lens for examining how nursing perspectives become visible or marginalised in governance processes. The value of the concept at this stage is therefore primarily theoretical and developmental, with empirical testing still required.

At the same time, the concept should be taken forward carefully. The literature does not yet provide a sufficiently consistent empirical basis for claims about measurement readiness, intervention effectiveness or direct impact. Future research should refine the boundaries of the concept, examine its expression in diverse nursing leadership settings and develop indicators that are conceptually aligned with its strategic, relational, ethical and contextual dimensions [41, 42].

### 5.1. Implications for Nursing

The findings suggest that political astuteness warrants greater attention in nurse leadership development, management preparation and professional socialisation. However, the present analysis supports this implication primarily at the level of conceptual clarification rather than intervention effectiveness. Accordingly, the implications offered here should be understood as analytically informed directions rather than prescriptive conclusions.

### 5.2. Implications for Nursing Education

The concept analysis suggests that nursing education could more explicitly engage with the policy, governance and stakeholder dimensions of leadership, particularly because political knowledge and policy engagement are longstanding and contemporary concerns in nurses’ professional preparation [12, 13, 43]. This is especially important because nursing education reform is itself shaped by policy processes, stakeholder coordination and regulatory alignment [44]. Educational approaches that expose students and emerging nurse leaders to health policy processes, systems thinking, advocacy, negotiation and stakeholder engagement may help build some of the capabilities associated with political astuteness. However, the present study does not provide evaluative evidence on which educational strategies are most effective, and this remains an important area for future research.

### 5.3. Implications for Nursing Practice

For nursing practice and management, the analysis suggests that political astuteness may be particularly relevant in contexts of organisational change, interprofessional negotiation, service redesign and policy implementation. Nurse leaders frequently work in environments shaped by competing priorities, constrained resources and layered governance arrangements, and the concept provides a useful lens for understanding how leadership is exercised under such conditions. The reviewed literature suggests that nurses’ political engagement may be constrained by limited policy preparation, organisational cultures, workload pressures and the marginalisation of the nursing voice in decision‐making spaces [7, 8, 39–41]. These constraints suggest that leadership development and organisational support structures may need to pay greater attention to the political and relational dimensions of nurse leadership practice.

### 5.4. Limitations

Several limitations should be considered when interpreting this concept analysis. First, although the included literature spans multiple countries, the evidence base is weighted toward high‐income Anglophone contexts, which may limit transferability across different health system, cultural and governance environments. Second, the body of literature includes a mixture of empirical, conceptual and theoretical sources, with relatively limited intervention‐based evidence. This means that the analysis is better positioned to clarify the concept than to establish the effectiveness of particular educational or leadership strategies. Third, some empirical referents rely on self‐report measures and related proxy constructs, which do not fully capture the broader conceptualisation of political astuteness advanced in this paper. Finally, the analysis focuses specifically on political astuteness in nurse leadership and does not attempt a broader comparison with all leadership effectiveness frameworks. Future research should therefore test the concept in diverse settings and work toward more contextually sensitive and conceptually aligned measures.

## 6. Conclusion

This concept analysis clarifies political astuteness as a multidimensional leadership capability relevant to nurse leadership in organisational, interprofessional and policy contexts. The findings suggest that political astuteness extends beyond interpersonal influence, general political awareness or policy advocacy and includes contextual reading, strategic judgement, stakeholder alignment and ethically informed action in complex governance environments.

By clarifying its defining attributes, antecedents, consequences and empirical referents, this study provides a more coherent conceptual foundation for understanding political astuteness in nurse leadership. Its main contribution lies in conceptual clarification rather than in demonstrating intervention effectiveness, measurement readiness or causal outcomes. In this sense, the analysis helps position political astuteness as a potentially useful concept for future nursing leadership scholarship, educational inquiry and professional development.

The study also highlights the need for further empirical and methodological work. Future research should examine how political astuteness is expressed in diverse nursing leadership settings, refine its conceptual boundaries and develop contextually sensitive and conceptually aligned indicators. Greater conceptual and empirical attention to political astuteness may strengthen understanding of how nurse leaders engage with policy, exercise influence and navigate the complexity of contemporary health systems.

## Author Contributions

Namadzavho Joyce Muswede conceptualised the research, critically reviewed the manuscript, made substantial revisions, and developed the case studies. Vhothusa Edward Matahela conceptualised the research, conducted the initial literature review, and conducted the concept analysis.

## Funding

This study did not receive any funding.

## Disclosure

All the authors reviewed and approved the final manuscript.

## Ethics Statement

Ethics approval and consent to participate were not required because this study was a concept analysis of published literature and did not involve human participants, identifiable personal data or primary data collection.

## Conflicts of Interest

The authors declare no conflicts of interest.

## Data Availability

All data used to support this concept analysis were derived from the published literature cited in this article.
